# Requirement for etoposide in the treatment of pregnancy related hemophagocytic lymphohistiocytosis: a multicenter retrospective study

**DOI:** 10.1186/s13023-019-1033-5

**Published:** 2019-02-18

**Authors:** Yue Song, Zhao Wang, Zengping Hao, Lihong Li, Junli Lu, Hongjun Kang, Yanping Lu, Yanqin You, Lijuan Li, Qingyun Chen, Bo Chen

**Affiliations:** 1 Department of Hematology, Beijing Friendship Hospital, Capital Medical University, YongAn Road 95th Xicheng District, Beijing, 100050 China; 2 Department of Obstetrics and Gynecology, Beijing Friendship Hospital, Capital Medical University, Beijing, China; 3 Department of Hematology, Beijing Chao-Yang Hospital, Capital Medical University, Beijing, China; 4 Department of Obstetrics and Gynecology, Beijing Chao-Yang Hospital, Capital Medical University, Beijing, China; 5 Department of Hematology, Chinese People’s Liberation Army General Hospital, Beijing, China; 6 Department of Obstetrics and Gynecology, Chinese People’s Liberation Army General Hospital, Beijing, China; 7 Department of Intensive Care Unit, China-Japan Friendship Hospital, Beijing, China; 8 Department of Obstetrics and Gynecology, China-Japan Friendship Hospital, Beijing, China

**Keywords:** Hemophagocytic lymphohistiocytosis, Pregnancy, Treatment option, Etoposide, Corticosteroids

## Abstract

**Background:**

Hemophagocytic lymphohistiocytosis (HLH) is a rare severe clinical syndrome. HLH manifesting during pregnancy has been paid much attention in recent years. Despite the specificity of pregnancy-related HLH, there has not been any consensus regarding its treatment. According to a previous study, corticosteroid/IVIG is the mainstream therapy; however, the efficacy is controversial. Etoposide is an important agent in the HLH-94 regimen; nevertheless, its use is limited because of possible toxicity to the fetus. Methods: In this study, we summarized 13 cases from 4 medical institutions from April 2011 to April 2018. Treatment regimens and outcomes were observed.

**Results:**

The median age was 26 (20–36) years old. The median gestational age was 28 (10–35) weeks. In these 13 patients, 10 were treated with methylprednisolone/IVIG and was effective in only two patients. In 6 patients who used etoposide during their treatment, all achieved remission. The median time from onset of disease to use of etoposide was 36 (17–131) days. Five of these 6 patients were treated with corticosteroids with/without IVIG before etoposide. One patient with pulmonary tuberculosis and one with lymphoma were treated according to etiology and achieved long survival.

**Conclusion:**

For treatment of pregnancy-related HLH, particularly for patients who do not respond to corticosteroids/IVIG therapy, etoposide should be used bravely. Nevertheless, suitable dosages and toxic and side-effects require further clinical observation.

## Background

Hemophagocytic lymphohistiocytosis (HLH) is a rare severe clinical syndrome that is characterized by dysregulated hyperinflammatory immune responses resulting in histiocytic proliferation with significant hemophagocytic activity in bone marrow and massive inflammatory cytokine release [1, 2]. This syndrome can be classified as either primary or secondary. Secondary HLH can be triggered by a variety of diseases, including infections, immunodeficiency syndromes, hematological malignancies and autoimmune diseases. Hemophagocytic lymphohistiocytosis manifesting during pregnancy is rare; nevertheless, in recent years, with deepening understanding, increasing attention has been paid to it. In a summary of cases reported from 1995 to 2017 [3], there was a total of 20 cases and half of them occurred during the latter 5 years. HLH is a severe clinical syndrome, developing rapidly without effective therapy with a very high mortality. The standard therapy for HLH is the HLH-94 regimen and sometimes the HLH-04 regimen [1]. Considering the specificity of pregnancy-related HLH: 1) it is rare clinically with lack of experience; and 2) there are high safety requirements for treatment; therefore, to date there has been no consensus regarding treatment. In previous studies, most patients were treated with corticosteroid/IVIG [4–9]. Patients with specific etiologies were treated according to the respective etiologies (e.g., B-cell lymphoma was treated with R-CHOP regimen [5], HSV infection was treated with acyclovir [10], HIV infection was treated with HAART regimen [11]) and cyclosporine A was only added in a few cases [8, 12]. It appears that corticosteroid/IVIG is the mainstream therapy for pregnancy-related HLH; however, the efficacy remains unclear. In a summary of 23 cases in the literature, there were 14 cases treated with corticosteroid and 9 were effective. Etoposide is a core is a chemotherapeutic agent in HLH-94 and HLH-04 treatment protocol [1, 2]; it is generally believed that it selectively inhibits the activation of monocyte-macrophage system by ablating overactivation of T cells, thereby reducing the generation of the inflammatory cytokine storm and controlling HLH [13]. Nevertheless, considering the possible toxicity to fetus and severe bone marrow suppression, etoposide has limited use in pregnancy-related HLH. Etoposide was used only in 5 cases reported in the literature to date, and 3 achieved remission after treatment. Comprehensive consideration is given to the relatively limited therapeutic effect of corticosteroid in pregnancy-related HLH and to the lack of effective therapeutic methods after ineffective corticosteroid therapy. Furthermore, most patients with pregnancy-related HLH have no clear etiology and therefore cannot be treated with respect to etiology. Therefore, etoposide may be a new direction of treatment for pregnancy-related HLH. We conducted a retrospective analysis of the effects of treatments methods of pregnancy-related HLH in several centers in China, and to study the requirement for etoposide these patients.

## Methods

### Subject eligibility criteria

Patients enrolled in this study fulfilled the following criteria: (1) the patient met HLH-2004 diagnostic criteria [1]; (2) the patient was pregnant at the time of onset.

### Assessment of therapy

The assessment of treatment was previously described in a research study for pediatric HLH. We modified the treatment based on our experience with adult HLH patients [14, 15]. A complete response was defined as normalization of all quantifiable symptoms and laboratory markers of HLH, including levels of sCD25, ferritin, triglycerides, hemoglobin, neutrophil counts, platelet counts and alanine aminotransferase (ALT). A partial response was defined as at least a 25% improvement in 2 or more quantifiable symptoms and laboratory markers by 2 weeks following DEP (doxorubicin-etoposide-methylprednisolone) regimen as follows: sCD25 response was > 1.5-fold decreased; ferritin and triglyceride decreased at least 25%; for patients with an initial neutrophil count of < 0.5 × 10^9^/L, a response was defined as an increase by at least 100% to > 0.5 × 10^9^/L; for patients with a neutrophil count of 0.5 to 2.0 × 10^9^/L, an increase by at least 100% to > 2.0 × 10^9^/L was considered a response; and for patients with ALT > 400 U/L, response was defined as an ALT decrease of at least 50%. Additionally, subject body temperatures had to revert to normal ranges in either complete response or partial response. Other observational indicators in the study included liver and spleen size, bilirubin and albumin levels.

The therapy regimens and outcomes were observed and documented.

## Results

### General characteristics

The data in this study were collected from 4 medical institutions in China: Beijing Friendship Hospital, General Hospital of the People’s Liberation Army, Beijing Chao-yang Hospital and China-Japan Friendship Hospital. From April 2011 and April 2018, there were 13 patients in total who were enrolled in this study. The median age was 26 (20–36) years old. The median gestational age was 28 (10–35) weeks. Seven patients were first-time pregnant and the rest were multiparas. Among these 13 patients, 2 had a history of abnormal pregnancy. Most HLH occurred in the mid- to late-gestation. None of these patients had family history of HLH.

### Associated factors

Some pregnancy-related HLH has a combination of etiological factors, including infections, rheumatism, lymphoma and so on. Four cases were associated with infection (two with EB virus infection, one with *Staphylococcus epidermidis* infection and one with *Mycobacterium tuberculosis* infection). One patient was diagnosed with angio-immunoblastic T-cell lymphoma via pathological biopsy of cervical lymph nodes 7 months after diagnosis of HLH. Two were associated with autoimmune disease (one had adult-onset Still’s disease and the other had systemic lupus erythematosus. The other 6 patients had no known associated disease.

### Therapy and outcomes

In these 13 patients, 10 were treated with corticosteroids (with/without IVIG) two were treated with methylprednisolone combined with cyclosporine A, and one was treated with HLH-94 regimen. 1) In these 10 patients treated with corticosteroids (with/without /IVIG), 2 were effective (partial remission). These 2 patients achieved complete remission after consolidation therapy of FD regimen (fludarabine and dexamethasone). Of the 8 patients who were not responding, one patient was diagnosed with tuberculosis, 4 patients had clinically improved but not biochemically, and 3 patients had HLH progression. The patient diagnosed with tuberculosis completely recovered after anti-tuberculous therapy. Five of the remaining 7 patients achieved remission (complete or partial remission) after receiving treatment with etoposide. One of them (case 3) re-fevered after 7 months and was diagnosed as immunoblastic T-cell lymphoma by pathological biopsy of cervical lymph nodes. After 3 courses of E-CHOP and allo-HSCT, she achieved complete remission. Case 4 relapsed after 20 days and was completely relieved after 3 courses of DEP regimen. The last 2 patients who did not respond to corticosteroids and did not receive other treatments subsequently died of HLH. 2) Of the two patients receiving corticosteroids combined with cyclosporin A, one patient achieved remission and the other patient was ineffective, but was relieved after the termination of pregnancy. 3) One patient who had a spontaneous abortion before treatment was treated with the HLH-94 regimen. The HLH-94 regimen was effective but HLH relapsed 1 month later. Lymphoma could not be excluded, but she refused further examination and treatment due to economic problems and subsequently lost her follow-up.

There were 6 patients who used etoposide during their treatment. The median time from onset of disease to use etoposide was 36 (17–131) days. Five were treated with corticosteroids with/without IVIG before etoposide. Cases 4 case 6 were treated with corticosteroids/IVIG and terminated pregnancy, but in neither case was treatment effective. Cases 1 and 3 were treated with corticosteroids/IVIG after delivery as their gestational ages were sufficient. Even though their temperature returned to normal, the abnormal laboratory findings worsened and they developed fever again in the short term (within 48 h or during the course of corticosteroid reduction). Case 5’s gestational age was 19 weeks; therefore, she was treated with corticosteroids during her pregnancy and did not achieve remission. Etoposide was effective in all these 6 patients. The temperature recovery time after etoposide was 24 h–5 d. Most of the patients’ temperatures returned to normal within 48 h. After 2 weeks of etoposide, the efficacy was evaluated, 4 patients of which reached CR and 2 of which reached PR.

As for the outcome of the fetus, 8 were delivered spontaneously or by c-section as the gestational age was adequate (28-36w). One of these 8 developed respiratory distress, and luckily survived with effective treatment. There were 4 fetuses were not mature enough (10-19w), then 2 suffered spontaneous miscarriage and 2 underwent induced abortion. Only 1 fetus died of respiratory distress as the mother developed HLH in 24w and the child had to be premature delivered. Only Case 5 had used etoposide during her pregnancy. The baby was delivered at term and currently healthy.

The follow-up ended on October 31, 2018 or the death of patients. Except for one patient who lost to follow-up, the mortality of the other 12 patients was 15.4% (2/12) and the median follow-up duration was 29 months (0.4–93 months). For the 6 patients who used etoposide, one lost to follow-up and the follow-up duration of the other 5 patients ranged from 5 to 43 months (median 27 months). All of these 5 patients lived. The clinical characteristics, treatment regimens and outcomes are summarized in Table 1.

**Table 1:** Characteristics of patient’s in this study with HLH during pregnancy

	Maternal age (years)	Gestational age (weeks)	Associated disease	Types of treatment	Response according to specified treatment	Survival Outcome of pregnancy	
						Maternal	Fetal
1	26	31	infection (*Staphylococcus epidermidis*)	Spontaneous delivery at 31w Corticosteroids, IVIG Etoposide	No improvement Complete remission	survive	alive
2	36	14	Unclear	Spontaneous miscarriage Corticosteroids, etoposide	Complete remission	defaulted	Dead
3	30	34	Angioimmunoblastic T-cell lymphoma (7 months later)	Spontaneous delivery at 34w Corticosteroids Etoposide Diagnosed of lymphoma: E-CHOP, allo-HSCT	No improvement Complete remission Complete remission	survive	alive
4	30	30	Unclear	Corticosteroids, IVIG C-section at 35w HLH-04 regimen DEP regimen	No improvement Partial remission Complete remission	survive	alive
5	27	19	Epstein-Barr virus	Corticosteroids Etoposide	No improvement Complete remission	survive	Alive
6	29	30	Unclear	Corticosteroids C-section (transverse lie) at 30w Etoposide	No improvement Complete remission	survive	Alive
7	24	10	Still’s disease	Corticosteroids, fludarabine Induced abortion at 16w	Complete remission	survive	Dead
8	24	17	Unclear	Corticosteroids, cyclosporine Induced abortion at 19w	No improvement Complete remission	survive	Dead
9	26	28	Tuberculosis	Delivered at 28w, Corticosteroids antituberculosis therapy	No improvement Complete remission	survive	Alive
10	20	10	Systemic lupus erythematosus	Corticosteroids, cyclosporine Spontaneous miscarriage	Complete remission	survive	Dead
11	24	36	Unclear	Delivered at 36w Corticosteroids, fludarabine	Complete remission	survive	Alive
12	29	28	Unclear	Corticosteroids Delivered at 28w	No improvement	Death	Alive
13	25	24	Epstein-Barr virus	Corticosteroids, IVIG Delivered at 24w	No improvement	Death	Dead (respiratory distress)

## Discussion

HLH is a macrophage proliferative disease involving multiple organs and systems that progressively aggravates with immune dysfunction. Its intrinsic etiology is such that it causes immune dysfunction by way of one or more factors, resulting in an inflammatory factor storm and a series of clinical manifestations. Pregnancy-related HLH, is very rare compared with the other types of HLH. Due to the rare clinical situation of pregnancy-related HLH, the lack of relevant experience and the need to consider the effect of drugs on the fetus during pregnancy, there is no unified clinical treatment recommendation at present. Summarizing the case reports in the literature between 1999 to 2017, there were 23 cases of pregnancy-related HLH (Table 2). Corticosteroid therapy was mostly used (60.9%, 14/23), and some were treated for the specific etiology, while a few were treated with cyclosporine A. Corticosteroids and IVIG are the main drugs for pregnancy-related HLH at present, and there was good response in previous reports (64.3%, 9/14). Nevertheless, in this study, the effect of corticosteroids/IVIG was not that clear (effective in 20%, 2/10), different from findings in the previous literature. The possible explanations for these findings include: 1) most of the patients in this study came from local hospitals, as they kept continued worsening in the local hospitals. Therefore, bias may exist in terms of evaluating the effect of corticosteroids/IVIG, because these patients observed in this study may suffer severer clinical courses. 2) of the 9 patients in the literature with effective use of corticosteroids, 4 were associated with autoimmune disease. In the present study, the 2 patients who were had associated autoimmune diseases responded well to corticosteroids and cyclosporine A. Nevertheless, the remaining 11 cases were associated with infection or with unclear etiology. The current treatment concept for MAS (macrophage activation syndrome, another name for the autoimmune disease associated HLH) is usually initiated: intravenous methylprednisolone pulse therapy; if response to steroids is not immediately stimulated, there is parenteral administration of cyclosporine A (CsA) (2–7 mg/kg/day) [16]. Therefore, there may be a possibility that in pregnancy-related HLH patients, those who were associated with autoimmune disease will response better to corticosteroids. Various disease spectra lead to varying therapeutic effects; 3) previous reports have suggested that the most common onset time of pregnancy-related HLH is the middle of pregnancy [3]. Therefore, corticosteroid therapy is often used to maintain pregnancy stability and take fetal factors into account. In this study, most of patients were in the later period of pregnancy and some of them had terminations due to factors such as sufficient gestational age or fetal distress. In previous studies, termination of pregnancy turned out to sometimes be effective as a treatment method (75%, 3/4, total 23 cases). In the present study, however, the termination of HLH was only effective in one patient. The reason for the differences is not clear, and may be related to the pathogenesis of HLH during pregnancy. There was a case report recently who developed HLH during her first and second pregnancies, and then she was found with a heterozygous UNC13D mutation. As this mutation has not yet been reported to cause familial HLH, it is likely that the mutation predisposed her to immune dysregulation/overactivation, and the pregnancy as a “trigger” resulted in the occurrence of HLH [17]. Even though there was no such phenomenon in our study, and no patients had suspicious family history of HLH, we still speculated that some women may have susceptibility genes or suspicious factors (such as infection, autoimmune disease) for HLH, while pregnancy is some kind of “trigger” or “high-risk factor” for the onset of HLH. This may be supported by the views that pregnancy represent an immunologically unique population whose immune system is dysregulated because of fetus recognition [18, 19]. So, termination of pregnancy may be effective in some cases. But, in this study, these patients’ inflammatory storms were so severe that removing “trigger” was not enough. That may be why the corticosteroids/IVIG did not perform well in this study. However, this is basically speculation and needs more detailed research.

**Table 2:** Characteristics of patient’s cases reported in the literature with HLH during pregnancy

	Maternal age (years)	Gestational age (weeks)	Associated disease	Clinical signs	Treatment	Outcome	Outcome
Nakabayashi et al. (1999) [29]	ND	21	Preeclampsia	Fever, hepatosplenomegaly, cytopenias, hyperferritinemia	IVIG	Complete remission	survive
Chmait et al. (2000) [30]	24	29	History of necrotizing lymphadenitis; EBV (discovered postmortem)	Fever, cytopenias, hyperferritinemia	Delivery	no response	Death
Yamagushi et al. (2005) [8]	ND	2nd trimester	HSV-2, genital herpes infection	Fever, skin lesions, pancytopenia, hypertriglycemia, hyperferritinemia	Corticosteroids; Cyclosporin A	Complete remission (failed corticosteroids, remission with Cyclosporin A)	survive
Hanaoka et al. (2007) [5]	33	23	B-cell lymphoma	Fever, hepatosplenomegaly, cytopenias, hyperferritinemia, hypertriglyceridemia, DIC, elevated sCD25	Emergent C-section (fetal distress); R-CHOP chemotherapy	Complete remission	survive
Perard et al. (2007) [6]	28	22	Systemic lupus erythematosus	Fever, pancytopenia, hypertriglycemia, hyperferritinemia	Corticosteroids, IVIG 3 doses, Premature Delivery	No improvement with steroids; premature delivery; complete remission after third IVIG dose	survive
Teng et al. (2009) [7]	28	23	Autoimmune hemolytic anemia	Fever, hepatosplenomegaly, Cytopenias, hyperferritinemia, hypertriglyceridemia	Corticosteroids Cesarean	Failed corticosteroids; complete remission after Cesarean	survive
Yoshida et al. (2009) [9]	ND	Post-partum	Systemic lupus erythematosus	Fever, cytopenias, hyperferritinemia hypertriglyceridemia	Corticosteroids	Complete remission	survive
Chien et al. (2009) [31]	28	23	Unclear	Fever, cytopenias, hyperferritinemia, hypertriglyceridemia	Cesarean delivery	Complete remission	survive
Arewa et al. (2011) [11]	31	21	HIV	Fever, jaundice, abdominal pain, cytopenias	HAART Delivery at term	Complete remission	survive
Hannebicque Montaigne et al. (2012) [32]	21	29	Systemic lupus erythematosus	Fever, pancytopenia, hyperferritinemia, hypertriglycemia	Corticosteroids IVIG	Complete remission	survive
Dunn et al. (2012) [33]	41	19	Still’s disease	Fever, rash, cytopenias, hyperferritinemia, hypertriglyceridemia, elevated sCD25	Corticosteroids	Complete remission	survive
Shukla et al. (2013) [34]	23	10	Unclear	Fever, hepatosplenomegaly, cytopenia, hypertriglycemia, hyperferritinemia	Corticosteroids; spontaneous abortion	Failed steroids; complete remission after abortion	survive
Mayama et al. (2014) [35]	28	21	Parvovirus B19	Fever, cytopenias, hyperferritinemia, hypertriglyceridemia	Corticosteroids	Complete remission	survive
Goulding et al. (2014) [10]	27	23	HSV-2	Fever, cytopenias and hyperferritinemia	Corticosteroids, acyclovir	Complete remission	survive
Klein et al. (2014) [36]	39	30	EBV	Fever, hepatosplenomegaly, cytopenias, hyperferritinemia	Corticosteroids, cyclosporine and etoposide in combination with Rituximab	no response	death
Tumian et al. (2015) [12]	35	38	CMV (postmortem diagnosis)	fever, cytopenias, hyperferritinemia, hypertriglyceridemia, hepatitis	Corticosteroids IgIV, cyclosporine	no response	death
Samra et al. (2015) [4]	36	16	Unclear	fever, hepatosplenomegaly, cytopenias, hyperferritinemia, hypertriglyceridemia,	Corticosteroids	Complete remission	survive
Rousselin et al. (2015) [37]	44	30	Autoimmune disease	Fever, hepatomegaly cytopenias, hyperferritinemia, hypertriglycemia	glucocorticoides	Complete remission	survive
Giard et al. (2016) [38]	35	13	Kikuchi Fujimoto lymphadenitis	fever, cytopenias, hyperferritinemia, hypertriglycemia	Corticosteroids Etoposide Abortion	response	death
Ikeda et al. (2017) [39]	32	11	EBV	fever, cytopenias, hyperferritinemia, hypertriglyceridemia	Single dose dexamethasone Etoposide Cyclosporine	Complete remission	survive
Robert et al. (2017) [40]	33	22	unclear	fever, hyperferritinemia	Corticosteroids, etoposide, BMT	Partial remission after etoposide, complete remission after BMT	survive
Yildiz et al. (2017) [3]	36	29	unclear	fever, cytopenias, hyperferritinemia hypertriglyceridemia	Corticosteroids	Complete remission	survive
He M et al. (2017) [41]	27	30	NK/T cells lymphoma	Fever, splenomegaly, cytopenias, hyperferritinemia	Corticosteroids and etoposide in combination with rituximab	no response	death

Etoposide is a chemotherapy medication used for the treatments with a number of cancers. It forms a ternary complex with DNA and the topoisomerase II enzyme (that aids DNA unwinding), preventing re-ligation of the DNA strands, and in so doing causes DNA strands to break. This causes errors in DNA synthesis and promotes apoptosis of the cancer cell [20]. In recent years, etoposide has been widely used in the treatment of histiocyte diseases. As one of the essential drugs in the HLH-94 and HLH-04 regimens, it is believed that etoposide acts through selective ablation of hyperactivated T cells, to inhibit the activation of monocyte-macrophage system and reduce the generation of the inflammatory cytokine storm, finally controlling HLH without damaging static and memory T cells [13]. This differs from the wide immunosuppression effects of corticosteroids and the immunoregulation of IVIG. The limitation of etoposide in pregnancy-related HLH is the possible harm to fetus and intense bone marrow suppression. Etoposide is AU TGA pregnancy category D and US FDA pregnancy category D, which means: there is positive evidence of human fetal risk based on adverse reaction data from investigational or marketing experience or studies in humans, but potential benefits may warrant use of the drug in pregnant women despite potential risks [21]. Even though etoposide exposure in 6 cases of previous literature during the second and third trimesters did not seem to cause any congenital malformations, concern still exists [22–25]. In this study, for the one patient who used etoposide during her pregnancy, no congenital malformations could be found in the fetus. However, in a recent study in mice, the results indicate the potential for adverse effects on fetal ovarian development [26].

In the previous 23 cases in the literature, etoposide was used in 5 cases (21.7%, 5/23), among which 3 patients improved after treatment. In this study, we clearly found that when corticosteroids/IVIG or other treatments cannot control HLH, etoposide’s effect was remarkable, quick and produced at least partial remission early; in the cases without further treatment (including transplantation and chemotherapy, etc.), as opposed to other types of HLH (EBV-HLH, LAHS, etc.) [27], the recurrence rate was low and the long-term prognosis was good. Given that etoposide may have certain side effects on the fetus, it is recommended that in the treatment of pregnancy-related HLH, the frequency of use of etoposide should be reduced to once a week for patients who are still pregnant. Due to the limited number of cases, further clinical studies are needed to clarify the side-effects of etoposide on the fetus and its relationship with dosage. For some reported concerns regarding myelosuppression, whether in the previous literature or in this study, etoposide prevented the development of cytopenia and marrow hypocellularity, presumably because its desirable effects on T cells were more potent that its off-target effects on marrow cells [13].

The appropriate timing of etoposide intervention needs more consideration. In the study of Imashuku et al., the probability of long-term survival was significantly higher when etoposide treatment was begun less than 4 weeks from diagnosis for patients receiving this agent later or not at all [28]. In this study, the median time of etoposide usage was 36 (17–131) days. Etoposide infusion was postponed given the possible side effects towards pregnancy and most cases start trying to use etoposide after the termination of pregnancy. In case 12 and 13, after the ineffective corticosteroids/IVIG, they didn’t get a chance to etoposide and finally died of HLH. However, no matter in previous literatures or this study, corticosteroids/IVIG is still effective in some cases. In which cases can the use of etoposide wait and in which cases do we need to use etoposide as early as possible? There’s still more work needed.

Some pregnancy-related HLH has a combination of etiological factors, including viral infections, rheumatism, lymphoma and others. Previous reports indicated that definitive treatment based on the etiology after the control of HLH was very important and effective, including R-CHOP for B-cell lymphoma, acyclovir for HSV infection and HAART for HIV infection; all these cases achieved remission and achieved long-term survival. In this study, of 2 patients who relapsed after remission, 1 was discovered to have a confounding factor (lymphoma) on further screening, and obtained long-term remission after lymphoma-specific treatment, and the other started screening for the etiology after the initial treatment was ineffective. Tuberculosis was explicit, and she achieved long-term survival after TB management. These conditions fully demonstrate the necessity of screening for related factors and their importance to treatment. Another patient with an EB virus infection died after treatment failure, corresponding to the poor prognosis of EBV-HLH. In a previous report, there were 3 cases of EB virus infection, of which 2 eventually died. A patient with NK/T-cell lymphoma also died after treatment failure, therefore, screening for relevant factors may also indicate the prognosis of pregnancy-related HLH. The overall prognosis of HLH in pregnancy is slightly better than that of other types of secondary HLH, and long-term remission is possible without transplantation when high-risk factors such as EBV are absent. In the previous 23 cases, the mortality rate was 21.7%. Two patients died in this study; 1 with CNS involvement. One patient had a clear complication of EBV infection and did not respond to corticosteroid therapy. After the disease progressed rapidly, the patient eventually died of the co-infection, related to the rapid progress and poor prognosis of EBV-HLH itself. In fact, most of the patients who died of the past 23 cases were associated with infection, especially EBV infection.

## Conclusion

HLH manifesting during pregnancy is rare in clinic. Achievement of balance of effective therapy and safety is critical. Etoposide is a classic drug for HLH treatment. Its effect in pregnancy-related HLH remains considerable. Especially for patients who do not respond to corticosteroids/IVIG therapy, etoposide should be used bravely. Nevertheless, more suitable dosages, toxicities and side-effects still require further clinical observation.
